# Reputation-Based Investment Helps to Optimize Group Behaviors in Spatial Lattice Networks

**DOI:** 10.1371/journal.pone.0162781

**Published:** 2016-09-09

**Authors:** Hong Ding, Lin Cao, Yizhi Ren, Kim-Kwang Raymond Choo, Benyun Shi

**Affiliations:** 1 School of Computer Science and Technology, Hangzhou Dianzi University, Hangzhou, 310018, China; 2 Key Laboratory of Complex Systems Modeling and Simulation, Ministry of Education,China, Hangzhou Dianzi University, Hangzhou, 310018, China; 3 Department of Information Systems and Cyber Security, University of Texas at San Antonio, San Antonio, TX 78249-0631, United States of America; 4 School of Information Technology and Mathematical Sciences, University of South Australia, Adelaide, 5059, Australia; University of Waterloo, CANADA

## Abstract

Encouraging cooperation among selfish individuals is crucial in many real-world systems, where individuals’ collective behaviors can be analyzed using evolutionary public goods game. Along this line, extensive studies have shown that reputation is an effective mechanism to investigate the evolution of cooperation. In most existing studies, participating individuals in a public goods game are assumed to contribute unconditionally into the public pool, or they can choose partners based on a common reputation standard (e.g., preferences or characters). However, to assign one reputation standard for all individuals is impractical in many real-world deployment. In this paper, we introduce a reputation tolerance mechanism that allows an individual to select its potential partners and decide whether or not to contribute an investment to the public pool based on its tolerance to other individuals’ reputation. Specifically, an individual takes part in a public goods game only if the number of participants with higher reputation exceeds the value of its tolerance. Moreover, in this paper, an individual’s reputation can increase or decrease in a bounded interval based on its historical behaviors. We explore the principle that how the reputation tolerance and conditional investment mechanisms can affect the evolution of cooperation in spatial lattice networks. Our simulation results demonstrate that a larger tolerance value can achieve an environment that promote the cooperation of participants.

## Introduction

In collaborative and distributed systems, such as Internet of Things (IoT) and Peer to Peer (P2P) networks, autonomous individuals cooperate with each other to accomplish relatively complicated tasks for their reciprocity targets. However, most individuals in such networks are rational, and pursue their selfish interest or gains. In other words, individuals in such systems may only prefer to obtaining services from, rather than providing services to, others [1, 2]. For example, in a P2P network, there exist individuals who are only interested in downloading (shared) resources but are reluctant to contribute any resources in return. Such individuals are known as free-riders, whose behaviors may result in degradation for the system performance, and have negative effect on the quality of experience of other individuals (especially, those individuals who have contributed resources but are unable to reap the associated benefits). If no mitigation mechanisms are taken, more individuals will stop contributing/sharing resources, and the system will suffer from the problem of *commons dilemma*, also known as the tragedy of the commons. In this case, incentive mechanisms for promoting cooperation among selfish individuals are one useful solution to enhance the smooth operation of such systems.

Evolutionary public goods game (PGG) has been widely used in the literature to model group decision and cooperation dynamics in many fields, such as biology, sociology, physics and computer science [3–5]. A typical *N*-player PGG involves *N* individuals, each of which decides whether to *cooperate* (*C*) or *defect* (*D*) as its strategy. A cooperator will contribute an investment into the public pool which represents for a cost to establish a service, while a defector will not contribute anything. The total investment in the public pool will be multiplied by a multiplication factor *r* (i.e., 1 < *r* < *N*), which represents the eventual benefits upon the completion of the service. Finally, the benefits will be equally distributed among all *N* players. Defectors in a public goods game play as free riders by sharing the same public goods as cooperators without any contribution. Such behaviors may ultimately lead to the tragedy of the commons.

In order to overcome the commons dilemma, extensive studies has focused on investigating the evolution of cooperation in complex networks, where individuals are forced to participate in all PGGs that centered on itself and its neighbors [3, 6], or optionally decide whether or not to participate a PGG [7], or unidirectionally select their group members [8]. Accordingly, a number of mechanisms have been proposed to promote cooperation behaviors, such as reputation [9], mobility [10, 11], punishment [12], rewards [13] and social diversity [14, 15]. Among them, reputation as a social engine of indirect reciprocity [16, 17], has been widely used to explain the high cooperation levels in human society. Existing research generally assumes that there is a common reputation standard accepted by all individuals, where an individual will be regarded as good (respectively, bad) if its reputation is larger (respectively, smaller) than a standard/threshold value. However, reputation is more or less a subjective value. Different individuals may have different evalation criteria (i.e., tolerance in this paper). Moreover, in a more realistic scenario, the reputation value should be bounded, and can be dynamically updated according to individuals’ historical behaviors in an evolutionary game.

In this paper, we propose a reputation-based investment model that takes into account both *bounded reputation* and *reputation tolerance* among individuals during the evolutionary process of a public goods game. By allowing for differences in reputation tolerance, a cooperative individual will contribute investment into the public pool only when the number of individuals with higher reputation is not less its tolerance value. In this case, the situation where all individuals possess the same tolerance value is regarded as a special case in our model, and generally exists in a collaborative environment. We simulate our model in a homogeneous spatial lattice network to demonstrate how the reputation-based model can significantly promote cooperation among selfish individuals. It is interesting to observe that a larger tolerance value can make for a better environment that favors the prevalence of cooperative behaviors. We also explore how the initial conditions (including the reputation distribution and the upper bound of reputation value) can influence the evolutionary rate of cooperation in the spatial public goods game.

The rest of this paper is organized as follows. In the next section, we introduce related studies about the evolution of cooperation. Then, we present our reputation-based model and evaluate the performance in a spatial lattice network setting. Finally, we summarize our findings and discuss potential implications of our proposed model.

## Related Literature

In the literature, there are a number of incentive mechanisms designed to promote cooperation behaviors in both collaborative and distributed applications, such as Ad Hoc networks [18], cellular networks [19] and wireless networks [20]. Efficient incentive mechanisms include kin selection [21, 22], direct reciprocity [23], indirect reciprocity [16, 24], group selection [25], punishment [12, 26], and rewards [13, 27]. Reputation has also been identified as one of the typical mechanisms of indirect reciprocity, where the evolution of cooperation relies on individuals’ mutual monitoring and assessments [28]. Specifically, indirect reciprocity describes a kind of phenomenon where two individuals behavior toward each other is based on their past behaviors. Suppose that individual *A* acted positively to individual *B*, then individual *B* would assign a high reputation score for individual *A*. Meanwhile individual *C*, who witnessed the positive behavior of individual *A* towards individual *B*, would remember individual *A*’s good performance (i.e., high reputation) and cooperate with individual *A* with a high possibility. In reality, one’s reputation cannot infinitely increase or decrease due to bounded rationality [29], therefore, the reputation values should be in a reasonable interval (i.e., bounded reputation in this paper).

Reputation-based models have been extensively studied under the condition of public information, where all individuals share the same reputation information. For example, Nowak et al. introduced an image scoring to quantify individuals’ reputation, where an individual’s image scoring increases when the individual donates to others by comparing their image scoring [24]. However, in more general and realistic situations, one should take into consideration private information, where individuals have different perception about other individuals’ reputation. Uchida showed that private information can lead to mismatches between the opinions of individuals even when they share the same moral compass [30]. In another work, Uchida and Sigmund investigated the competition of the assessment rules for indirect reciprocity, where individuals assess each other according to their past cooperative behaviors [31]. Both public and private information determine how individuals dynamically perceive reputation of each other over time, while private information can better simulate the model of reputation in structured populations. Taking into consideration errors of information dissemination, Wang et al. investigated how different information about others individuals’ contributions affects conditional cooperators’ willingness to cooperate in a one-shot linear PGG [32]. In this paper, we introduce a reputation-based model, where individuals contribute investment based only on the private knowledge about the number of cooperators in the game.

There have also been extensive studies on the role of diversity (e.g., social diversity [3, 6], structural diversity [33–35], and behavioral diversity [36, 37]) in the evolution of cooperation. Spatial reciprocity is identified as one of the most effective means to enhance the cooperation levels, where social behaviors of individuals are modeled by the classical and evolutionary game theory in spatially structured populations [38–41]. For example, in a setting where individuals are coupled in a spatial network with interactions restricted to only their neighbors, cooperators can sustain by forming compact clusters in order to resist the exploitation of defectors [42]. Hartig et al. studied PGGs on square lattice and scale-free networks, and determined that not only the diversity of the number/size of PGGs, but also the diversity of individuals’ donation to every group member help to promote cooperation behaviors [43]. Accordingly, in this paper, the performance of a reputation-based investment model will be simulated in spatial lattice networks.

With respect to individuals’ behavioral diversity, investment heterogeneity is also a critical factor in reputation studies [7, 44]. Different levels of reputation heterogeneity may significantly influence the evolution of cooperation. For example, Helbing et al. investigated the impact of reputation-based investment heterogeneity on the evolution of cooperation in collaborative networks [45]. Tian et al. proposed an age-related preferential selection mechanism to study the impact of aging on the evolution of cooperation in the Prison’s dilemma game [35]. Specifically in this paper, an individual will determine whether or not to contribute investment into the public pool based on the individual’s private and heterogeneous tolerances about their neighbors’ reputation in a spatial lattice network.

## Reputation-based Investment Model

In this section, we present a reputation-based investment model in a public goods game, and investigate the evolutionary dynamics of cooperation in a spatial lattice network.

### Basic model

In a typical *N*-player PGG, individuals who cooperate are required to contribute some investment (i.e., cost of cooperation) into a public pool. Each individual knows that the total amount of investment in the public pool will be multiplied by a factor *r* (1 < *r* < *N*), and divided equally among them irrespective of their contributions. If all individuals cooperate, each of them will increase its initial capital by (*r* − 1)*c*, where *c* denotes the cost of cooperation. However, individuals are more likely to defect by exploiting other individuals’ investment without any cost. Obviously, such selfish behavior yields a higher payoff, irrespective of other individuals’ actions, because the investment of each cooperator returns only a fraction *r*/*N* < 1 of its investment. Therefore, although the group’s total payoff is maximized when all individuals cooperate, the Nash equilibrium in this game is simply zero contributions by all individuals.

In this paper, we focus on studying an evolutionary PGG in a spatially structured population, where individuals interact only with their immediate neighbors (known as *Von Neumann neighbors*). Specifically, each individual are confined in a square lattice, and restricted to play PGGs with its four neighbors. Formally, we denote *G*(*V*, *E*) as the square lattice with *M* nodes, where *v*_*i*_ ∈ *V* denotes the player *i* and *E*_*ij*_ ∈ *E* denotes the interaction between player *i* and *j*. Each player has exactly four neighbors in *G*(*V*, *E*). During the evolutionary process, each player *i* can dynamically change its strategy *s*_*i*_ (*s*_*i*_ ∈ {*C*, *D*}). Here, *C* strategy means a player will cooperate with others, while a *D* strategy means a player will defect.

The proposed reputation-based investment model is built upon the evolutionary PGG in *G*(*V*, *E*), where each round consists of two main phases, namely, an *investment phase* and a *learning phase*. In the investment phase, each player interacts with his four nearest neighbors, and totally participates in five PGGs (one is centered at itself and the other four PGGs are centered at its four neighbors). Different from typical PGGs, here a cooperative player will conditionally contribute investment to the public pool based on other players’ reputation. During each round, all players will receive their accumulated payoffs when all PGGs are finished. In the learning phase, each player has chance to change strategy by imitating one of his/her neighbors. More details will be presented in subsequent sections.

### Reputation updating rule

A reputation mechanism is introduced to model such situation in this section. We assume that players do not operate in a full anonymity environment. In other words, players may collect information about their potential interaction partners from their neighboring environment. Further, we assume that the reputations of players are publicly known to individuals who play the same PGG, which is reasonable due to the limitation of individual player’s capability.

In order to efficiently record the strategy each individual adopts, we introduce a *reputation tolerance mechanism* to help players choose potential partners with high reputation. Initially, players in the network randomly adopt a strategy, either cooperate (*C*) or defect (*D*). During the interaction phase, players decide whether or not to contribute to the public pool based on their strategies. Cooperative players contribute conditionally to the public pool, while defective players will never contribute. As for a cooperative player *i* in a PGG, he/she will first count the number of group members whose reputation are equal or greater than his own in the game. If the number exceeds or equals to his/her pre-determined *reputation tolerance* *T*_*i*_, the cooperator will contributes one unit to the public pool. We denote *s*_*i*_ as the strategy of player *i*, where *s*_*i*_ = 0 represents the cooperative strategy and *s*_*i*_ = 1 represents the defective strategy. Then, player *i*’s action *a*_*i*_ can be denoted as:

$$a_{i}=\left\{\begin{array}{cc} 0 & \text{if}\;s_{i}=0\;\text{and}\;n_{r_{i}}\geq T_{i}\\ 1 & \text{if}\;s_{i}=1\;\text{or}\;(n_{r_{i}}<T_{i}\;\;\&\;s_{i}\;=\;0)\;, \end{array}\right.$$ (1)

where *n*_*r*_*i*__ is the number of other players in the game whose reputation is not less than *i*’s reputation *R*_*i*_. Here, *a*_*i*_ = 0 denotes player *i* will contribute, and *a*_*i*_ = 1 denotes a non-contributing player *i*.

During the evolutionary process, the reputation of cooperative players who contribute investment to the public pool will be increased by one unit, whilst the reputation of players who benefit without contributing will be reduced by one unit. We assume that the players’ reputation is bounded in a region [0, *θ*]. In other words, if a player’s reputation exceeds *θ*, his/her reputation will be capped at *θ*. Similarly, a player’s reputation will never drop below 0. The updating rule for player *i*’s reputation at evolutionary round *t* can be described as follows:

$$R_{i}\left(t\right)=\left\{\begin{array}{cc} 0 & \text{if}\;R_{i}\left(t-1\right)=\theta \;\;\&\;a_{i}\;=\;0\;\\ 1 & \text{if}\;R_{i}\left(t-1\right)=0\;\;\&\;a_{i}\;=\;1\;\\ R_{i}\left(t-1\right)+{\left(-1\right)}^{a_{i}} & \text{if}\;0<R_{i}\left(t-1\right)<\theta , \end{array}\right.$$ (2)

where *R*_*i*_(*t*) represents player *i*’s reputation at round *t*.

### Investment and payoff

In the spatial lattice network *G*(*V*, *E*), each player will participate in five PGGs. In each PGG, the total amount of investment in the public pool is multiplied by the multiplication factor *r*, and then be distributed equally to all group members irrespective of their strategies. If no cooperator contributes, then no payoff will be distributed. Therefore, the payoff of a player *i* in each PGG will be given by:

$$p_{i}=\left\{\begin{array}{cc} 0 & \text{if}\;n_{c}=0\\ \frac{rn_{c}}{5}-1+a_{i} & \text{if}\;n_{c}>0, \end{array}\right.$$ (3)

where *n*_*c*_ is the number of cooperators who contributes to the public pool in the PGG.

### Learning rule

At each evolutionary round, all players will synchronously update the strategies according to a learning rule. For each player *i*, he/she randomly selects a player *j* from his/her four neighbors, and then adopt *j*’s strategy based on the following Fermi function:

$$W_{i\to j}=\frac{1}{1+exp[-\left(P_{j}-P_{i}\right)/K]},$$ (4)

where *P*_*i*_ represent player *i*’s accumulated payoffs after participating in five PGGs in his/her neighboring environment. *K* indicates the amplitude of noise, which quantifies the force of selection about strategy adoptions [46, 47]. If *K* → ∞ (weak selection), players are less responsive to payoff differences, and a player with high payoff may adopt the strategy of a less successful one. If *K* → 0, then players reliably switch to the strategy with the higher payoff even if the difference is very small. For 0 < *K* < ∞, there exists a relatively certain possibility that strategies with less payoffs will be adopted. In general, egoistic players prefer to adopt the strategy of more successful neighbors [48–50].

**Algorithm 1:** The Main Algorithm

**1** Generate a spacial lattice network *G* of size *M*

**2** Initialize strategies *s*_*i*_ for each individual *i*

**3** Initialize model parameters *T*, *K*, *θ*, *r* and *μ*

**4** **foreach** *iteration t* **do**

**5**  Setting the initial payoff of each node as zero

**6**  **foreach** *node i* ∈ *G* **do**

**7**   Play PGGs with its four neighbors

**8**   **if** *s*_*i*_ = 1 **then**

**9**    *a*_*i*_ = 1, i.e., *i* does not contribute to the pool

**10**   **else**

**11**    **if** *n*_*r*_*i*__ ≥ *T* **then**

**12**     *a*_*i*_ = 0, i.e., *i* contribute to the pool

**13**    **else**

**14**     *a*_*i*_ = 1, i.e., *i* does not contribute to the pool

**15**   Calculate the cumulative payoff for each node in the PGGs

**16**  **foreach** *node i* ∈ *G* **do**

**17**   Update *s*_*i*_ based on Eq 4

**18**   Generate a random value *x* ∈ [0, 1]

**19**   **if** *x* ≤ *μ* **then**

**20**    $s_{i}=\bar{s_{i}}$

**21**  Reset all nodes’ payoffs to zero

**22** Record the frequency of cooperation for each iteration

In order to better simulate real-world situations, we introduce strategic mutations in our model, where a cooperator is likely to become a defector, and vice versa. For simplicity, mutation only happens at the end of each round with a constant rate *μ*. That is to say, each player *i* has chance to change his/her strategy as follows:

$$s_{i}=\left\{\begin{array}{cc} \bar{s_{i}} & \text{with}\;\text{probability}\;\mu \\ s_{i} & \text{with}\;\text{probability}\;1-\mu , \end{array}\right.$$ (5)

where $\bar{s_{i}}$ denotes an opposite strategy of *s*_*i*_. Player *i* will change to another strategy with probability *μ*, or remains unchanged with probability 1 − *μ*.

In summary, the main procedure of the reputation-based investment model is shown in Algorithm 1.

## Experiments and Results

Simulations were carried out on a square lattice network of size *M* = 100 × 100 with periodic boundary conditions. Initially, cooperators and defectors were randomly distributed among the network with equal probability 50%). The key quantity to characterize the cooperative behavior is the cooperation frequency, which is defined as the ratio of the number of cooperator *M*_*c*_ to the total number of players *M* at the steady state (i.e. $\rho_{c}=\frac{M_{c}}{M}$). We aim to evaluate the effects of reputation tolerance *T*, multiplication factor *r*, selectoin strength *K*, maximum reputation *θ* and initial distribution of strategies on the evolution of cooperation in a PGG. All simulations are carried out over 1,000 Monte Carlo time steps, and we have tested that steady state can be reached.

### Effects of reputation tolerance and multiplication factor

**Fig 1:**
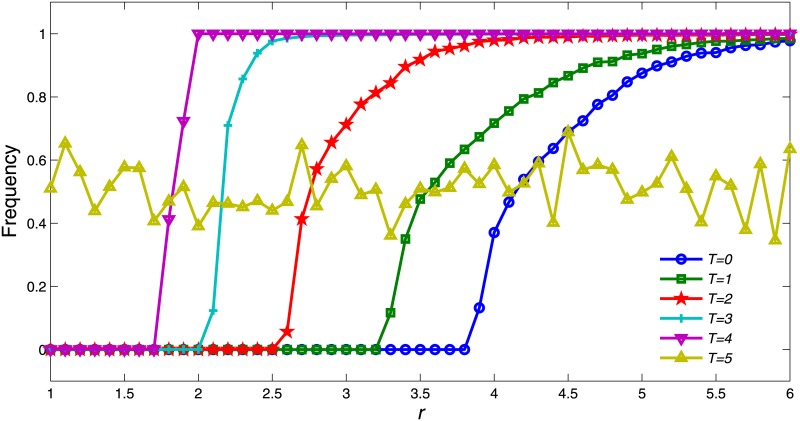
Cooperation frequency *ρ*_*c*_ acts as a function of *r* for different reputation tolerance *T*. Each data point results from the average value of the proportion of cooperators for the last 100 rounds after reaching steady state. The other parameter settings are *θ* = 20, *K* = 0.5 and *μ* = 10^−4^.

We first investigated the dependence of multiplication factor *r* with a stationary *T*. Fig 1 shows the average proportion of cooperators as a function of *r* ranging from 1 to 6, for different values of *T*. It can be observed that as cooperation frequency increases, so does *r*. Here, *T* is a set of six integers, namely: 0, 1, 2, 3, 4 and 5. *T* = 0 indicates that a cooperator will contribute even if all remaining group members’ reputation are less than himself (i.e., the player will contribute unconditionally). *T* = 5 indicates that a cooperator will not contribute to the public pool under any circumstances because it is impossible to have five players with a reputation higher than the particular cooperator in a five-member group. In the situation *T* = 0, where every cooperator contributes unconditionally, defectors who obtain more payoff easily occupy the lattice; thus, the final probability of cooperators is almost 0 under a moderate multiplication factor *r*. In *T* = 5 since no cooperators contribute, the probability of cooperators is influenced merely by mutation. Thus, the probability of cooperators fluctuating is around 0.5, regardless of the size of *r*.

With the increase in *r*, there is a phase transition in terms of cooperation frequency for each tolerance value of *T* = 0, 1, 2, 3, 4. It can be observed that for *T* = 4, the phase transition will happen when *r* is small (i.e., around 1.7). As *T* decreases, the critical value of *r* increases accordingly. When *T* = 0, the probability of cooperators frequency does not exceed 0 until *r* reaches approximately 3.9 and increases slowly to 1. The most prominent scenario is when *T* = 4, where a cooperator would not contribute unless his/her reputation is the smallest among all players in the PGG; thus, the probability of cooperators undergoing an abrupt increase when *r* is between 1.6 and 1.9. It can be observed from Fig 1 that the larger *T* is, the more promptly it leads to the cooperation level reaching a plateau for large values of *r*, except for *T* = 5.

**Fig 2:**
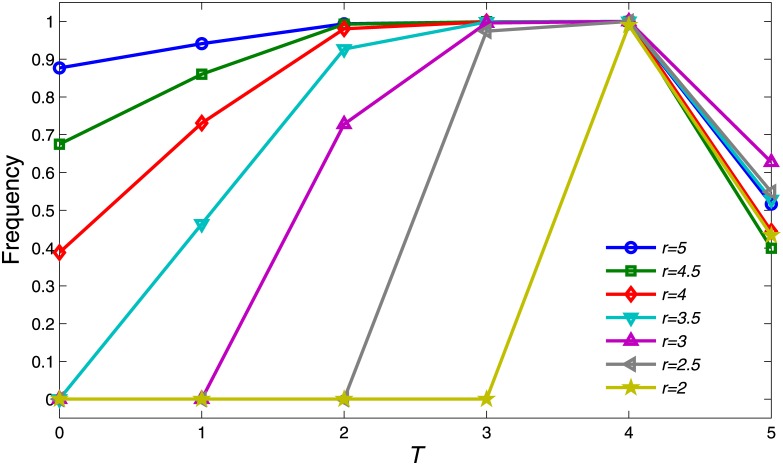
Cooperation frequency *ρ*_*c*_, as a function of *T* for different values of *r*. Each data point resulted from average value of the frequency of the proportion of cooperators for the last 100 rounds after reaching steady state. The other parameter settings are *θ* = 20, *K* = 0.5 and *μ* = 10^−4^.

To further investigate how the growth of *T* will influence the frequency of cooperators, we conducted experiments with different values of *r*. It can be observed from Fig 2 that when *T* = 4, the network will reach a high level of cooperation (i.e., almost 1) for different values of *r*. Only sufficiently large *r* can promote cooperation frequency when *T* = 1. When *T* = 2, 3, there exists a plateau of high level of cooperation. The results indicate that in order to resist the exploitation of defectors, it is more important for cooperators to contribute only when others’ reputations are no less than his own.

**Fig 3:**
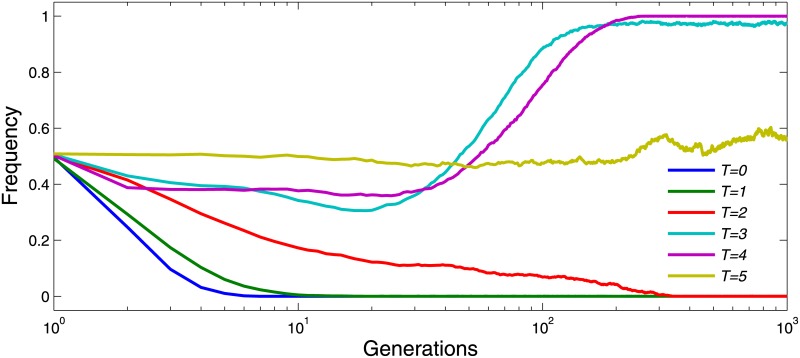
Cooperation frequency *ρ*_*c*_ with *r* = 2.5. Every colored solid line describes the evolution of the frequency of cooperators in 1,000 generations. Of the six reputation tolerance levels, *T* = 3 and 4 resulted in high cooperation. The other parameter settings are *θ* = 20, *K* = 0.5 and *μ* = 10^−4^.

To understand the process of how cooperation evolves, Fig 3 shows the change of cooperation frequency with respect to a fixed multiplication factor *r* = 2.5. Cooperation frequency decreases at the earlier generations, regardless of the value of *T* (with the exception of *T* = 5, which is an impossible situation). It is because the cooperators are distributed randomly in the lattice initially, and they could hardly survive due to the exploitation of defectors who can achieve high payoffs and whose behaviors will be imitated by others. However, individuals gradually congregate to compact clusters, among which cooperative clusters will result in a plateau of high cooperation frequency when *T* = 3 and 4. While defectors could exploit all cooperators when *T* = 2 and 3. The results demonstrate that low tolerance to others’ reputations can benefit the construction of cooperative environment.

**Fig 4:**
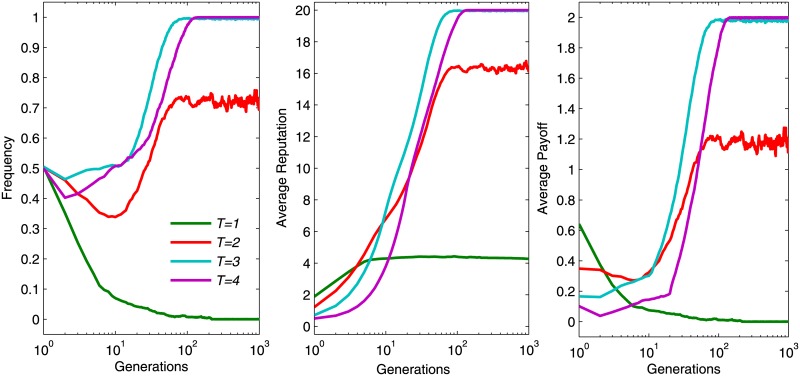
Cooperation frequency *ρ*_*c*_ (the left) corresponding average reputation (the center) and relevant average payoff (the right) of all population when *r* = 3. Every colored solid line describes how the parameters evolve for 1,000 generations. The other parameter settings are *θ* = 20, *K* = 0.5 and *μ* = 10^−4^.

**Fig 5:**
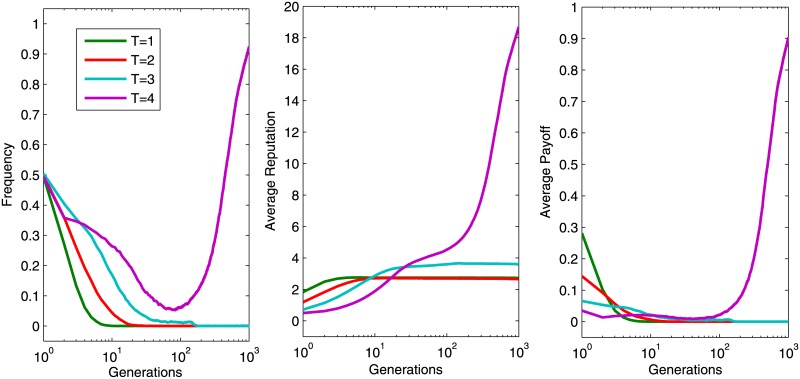
Evolution of cooperation frequency *ρ*_*c*_ (the left), average reputation (the center) and relevant average payoff (the right) of all population when *r* = 2. The other parameter settings are *θ* = 20, *K* = 0.5 and *μ* = 10^−4^.

We now investigated the evolution of individuals’ reputation, payoffs and cooperation frequency during the evolution. As shown in Fig 4, a larger *T* can significantly promote the evolution of cooperation. Cooperators are unable to escape the exploitation of defectors at the earlier stage of evolution. This resulted in a decrease of cooperation frequency and a moderate growth of the average reputation and payoff (expect for *T* = 1, where cooperation frequency decreases and the average reputation and payoff remain at a lower level). If the member’s *T* is large, then cooperators assembled faster than a small *T*, which manifested as their velocities to achieve stabilization are much swifter. The reason is that a larger *T* requires strict adherence to the group members that maintains high cooperation level in the entire population. The variation tendency of average reputation and payoff is similar to the frequency of cooperators. The maximum reputation is 20 and the maximum payoff is 2, which can be calculated by $\frac{r\cdot n_{c}}{5}-a_{i}$. Therefore, the optimal scenario is that all five group members adopt cooperative strategy with *a*_*i*_ = 1 and *r* = 3 in this experiment. Similar to the results demonstrated in Fig 4, the results presented in Fig 5 are conditioned on *r* = 2. We observed that the trend of cooperation frequency resembled the trend of average reputation and payoff. The average reputation and payoff have the same tendency as the frequency of cooperators globally. The cooperation frequency achieves a high cooperation level only when *T* = 4.

**Fig 6:**
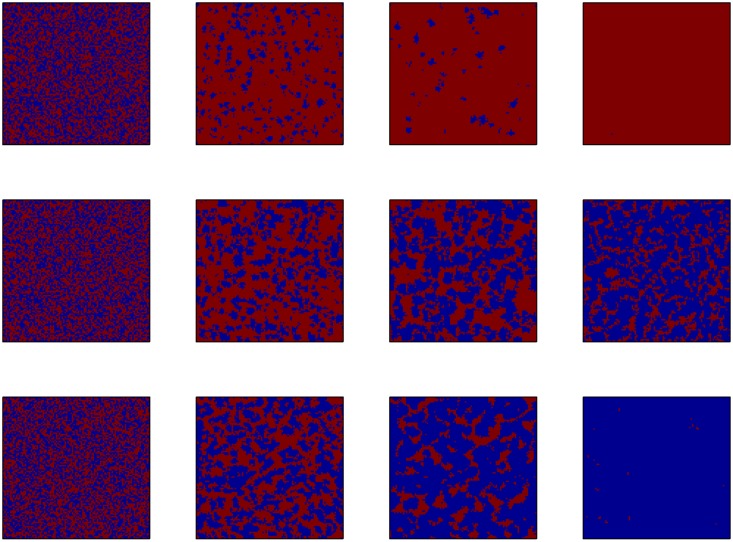
Snapshots of players’ strategies with parameters *r* = 2, *T* = 3 (the first row); *r* = 3, *T* = 2 (the second row) and *r* = 3, *T* = 3 (the third row). Blue pixels and red pixels represent the cooperative and defective strategy, respectively. The four columns show the results of the 1*^st^*, 10*^th^*, 30*^th^* and 10000*^th^* generations. The other parameter settings are *θ* = 20, *K* = 0.5 and *μ* = 10^−4^.

To further illustrate the competition between cooperators and defectors in a spatial lattice, in Fig 6, we evaluated the spatial distribution of defectors and cooperators under different parameter settings. The frequency of cooperators may undergo the exploration of defectors around the 10*^th^* generation. As more cooperators survive from the exploitation, the remaining cooperators gradually formed small clusters. This would allow them to swiftly expand to the entire lattices.

### Effects of maximum reputation and its initial distribution

**Fig 7:**
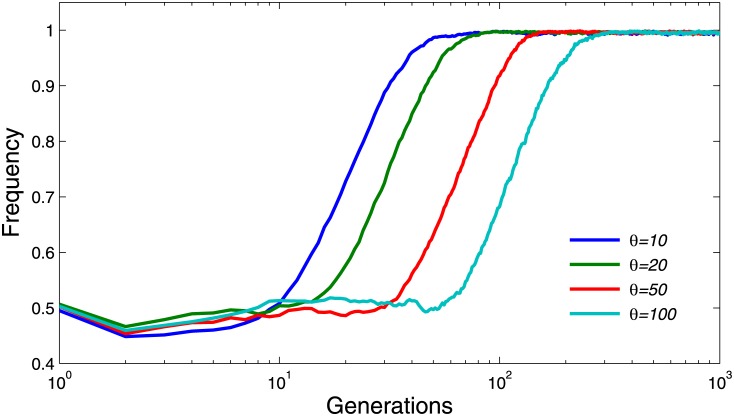
Evolutionary dynamics of cooperation frequency for different values of maximum reputation *θ* when *T* = 3 and *r* = 3. The other parameter settings are *K* = 0.5 and *μ* = 10^−4^. As shown in the Figure, the higher the maximum reputation is, the faster the evolution will converge.

**Fig 8:**
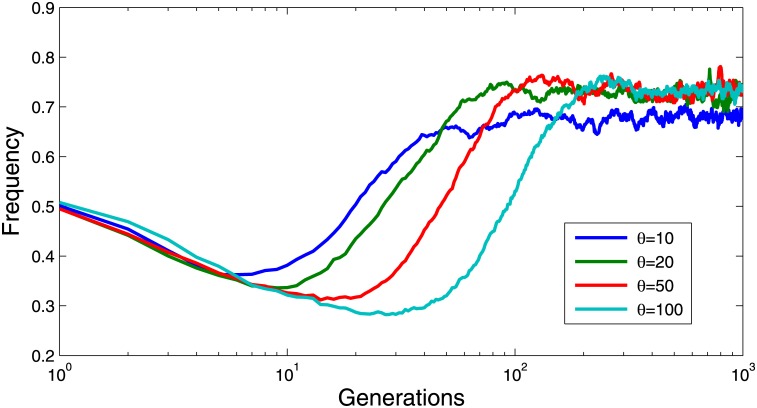
Evolutionary dynamics of cooperation frequency for different values of maximum reputation *θ* when *T* = 3 and *r* = 2. The other parameter settings are *K* = 0.5 and *μ* = 10^−4^.

In this section, we investigated whether the maximum reputation have any effects on the evolution of cooperation. Recall that in our model, the reputation of a cooperator who contributed to the public pool will be increased by one unit, while the reputation of players who benefit but without contributing will be reduced by one unit. The reputation value ranges from 0 to a maximum value *θ*. If one player’s reputation exceeds the maximum value, then his reputation will be capped. It can be observed from Figs 7 and 8 that the higher the maximum reputation is, the faster the evolution will converge. However, the values of *θ* will not affect the final cooperation frequency at the steady state. Different from the results in Fig 7, it can be found from Fig 8 that higher maximum reputation may result in better and more stable cooperation frequency.

**Fig 9:**
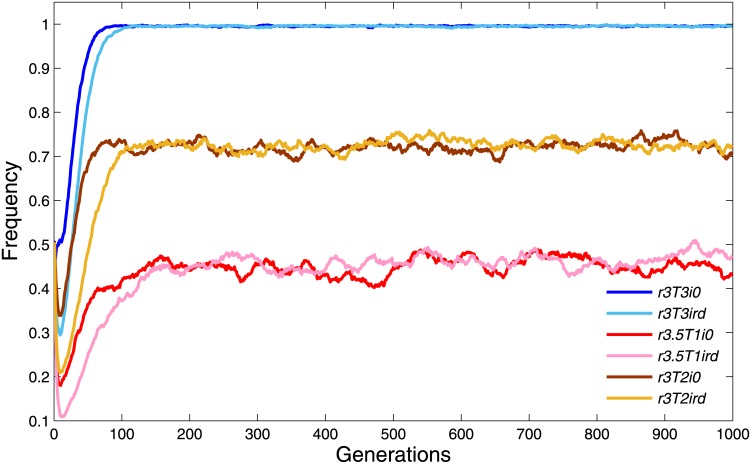
Evolutionary dynamics of cooperation frequency under different initial settings. The two blue lines show the results when *r* = 3 and *T* = 3; the two reds lines show the results when *r* = 3.5 and *T* = 1; and the two yellow lines shown the results when *r* = 3 and *T* = 2. For darker colored lines, all individuals’ initial reputation values are set to be 0, while for lighter colored lines, individuals’ initial reputation values are randomly selected from [0, *θ*]. The other parameter settings are *θ* = 20, *K* = 0.5 and *μ* = 10^−4^.

In Fig 9, we investigate the impact of different initial settings of reputation values on the evolution of cooperation frequency. Two types of settings are investigated. Firstly, all individuals’ reputation values are set to be zero, and secondly, the reputation values are randomly selected from [0, *θ*]. It can be observed that for the first setting, it is easier for cooperation frequency to reach a stable state. While for the second setting, it takes slightly more generations to reach a stable state. However, both settings do not affect the final results of cooperation frequency. The reason may be that if the reputation of all individuals is initially zero, then cooperative individuals are more likely to have high reputation. In doing so, it would be much easier to form a cooperative cluster in a spatial lattice network. On the contrary, if reputation values are randomly distributed among individuals, then it is possible for defectors to have a high initial reputation. Thus, it will take several generations to correct the reputation of those undeserving individuals. Therefore, under the second setting, the cooperation frequency is more unstable at the earlier generations.

### Effects of selection force *K*

**Fig 10:**
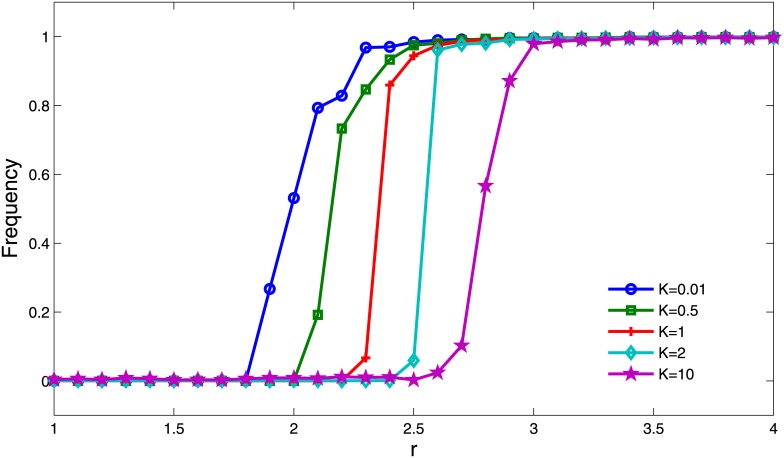
Cooperation frequency *ρ*_*c*_ as a function of *r* for different selection force *K*. The other parameter settings are *T* = 3, *θ* = 20 and *μ* = 10^−4^.

We also evaluated the effects of selection force *K* on the evolution of cooperation. It can be observed from Fig 10 that in the case of *K* = 0.01 (i.e., strong selection), the state of all defectors can only maintain for a very small value of *r*. In other words, the phase transition from all defectors to all cooperators will happen when *r* is small. While for *K* = 10 (i.e., weak selection), it can be observed that the phase transition of cooperation frequency happens for larger values of *r*. The reason is that the cooperators are more likely to survive by forming spatial clusters by mutually imitating behaviors of each other under a strong selection. On the other hand, once the cluster of cooperators is formed, the behaviors of cooperators are more easily imitated by defectors with a strong selection.

## Conclusion

In this paper, we proposed a reputation-based investment mechanism to investigate the evolution of cooperation in a spatial public goods game. The proposed mechanism has two properties, namely: each individual’s investment behavior is conditional based on other individuals’ reputation and its own reputation tolerance, and the reputation values are bounded.

We then evaluated the performance of the proposed mechanism in a spatial lattice network. The findings demonstrated that individuals’ reputation-based conditional investment behaviors can promote the evolution of cooperation for higher values of reputation tolerance. Specifically, if there are more individuals with strict reputation tolerance, then it will be easier to reach high the proportion of cooperators in the network (even with a small multiplication factor). This is due primarily to the spontaneous emergence of quarantine of defectors, who are eventually surrounded by cooperators and forced into isolated convex. This phenomenon can be observed in the simulation results on spatially structured populations. In addition, we investigated the effects of reputation tolerance, maximum reputation and selection force on the evolution of cooperation. Cooperation frequency initially decreased, as shown in the snapshots of several simulations for many reputation tolerance. In the long run, however, the evolution of cooperation ameliorated due to the formation of extremely robust clusters of cooperators. Moreover, the initial distribution of reputation appeared to have little influence on the evolution of cooperation, and only influencing the speed of convergence, where random reputation distribution converged faster. Our findings in this paper can help understand the role of reputation-based conditional investment in spatial public goods games.
